# Supplementary material for: A fault-intrusion-tolerant system and deadline-aware algorithm for scheduling scientific workflow in the cloud
Source: PeerJ Comput Sci. 2021 Nov 2;7:e747. doi: 10.7717/peerj-cs.747 (PMC8576554; doi:10.7717/peerj-cs.747)
Supplement: Supplemental Information 6 [file peerj-cs-07-747-s006.zip › Myproject1.1/docs/index-files/index-23.html]

Z-Index


JavaScript is disabled on your browser.


- Overview
- Package
- Class
- Use
- Tree
- Deprecated
- Index
- Help

- Prev Letter
- Next Letter

- Frames
- No Frames

- All Classes

A B C D E F G H I J L M N O P Q R S T U V W Z 


## Z

ZipfDistr - Class in org.cloudbus.cloudsim.distributions
:   The Class ZipfDistr.

ZipfDistr(long, double, int) - Constructor for class org.cloudbus.cloudsim.distributions.ZipfDistr
:   Instantiates a new zipf distr.

ZipfDistr(double, int) - Constructor for class org.cloudbus.cloudsim.distributions.ZipfDistr
:   Instantiates a new zipf distr.

A B C D E F G H I J L M N O P Q R S T U V W Z

- Overview
- Package
- Class
- Use
- Tree
- Deprecated
- Index
- Help

- Prev Letter
- Next Letter

- Frames
- No Frames

- All Classes
